# Effect of urban air pollution on CRP and coagulation: a study on inpatients with acute exacerbation of chronic obstructive pulmonary disease

**DOI:** 10.1186/s12890-021-01650-z

**Published:** 2021-09-18

**Authors:** Lingling Tang, Suofang Shi, Bohan Wang, Li Liu, Ying Yang, Xianhong Sun, Zhenhua Ni, Xiongbiao Wang

**Affiliations:** 1 Affiliated Hospital of Nanjing University of Chinese Medicine, Nanjing, 210029 Jiangsu China; 2 Department of Respiratory Medicine, Affiliated Hospital of Nanjing University of Chinese Medicine, Nanjing, 210029 Jiangsu China; 3 Department of Central Lab, Affiliated Hospital of Nanjing University of Chinese Medicine, Nanjing, 210029 Jiangsu China; 4 Department of Respiratory Medicine, Putuo Hospital, Shanghai University of Traditional Chinese Medicine, Shanghai, 200062 China; 5 Department of Central Lab, Putuo Hospital, Shanghai University of Traditional Chinese Medicine, Shanghai, 200062 China

**Keywords:** Acute exacerbation of chronic obstructive pulmonary disease, Ambient air quality, Particulate matter, Coagulation, Inflammation

## Abstract

**Purpose:**

Acute exacerbation of chronic obstructive pulmonary disease (AECOPD) is an important event in the course of chronic obstructive pulmonary disease that negatively affects patients’ quality of life and leads to higher socioeconomic costs. While previous studies have demonstrated a significant association between urban air pollution and hospitalization for AECOPD, there is a lack of research on the impact of particulate matter (PM) on inflammation and coagulation in AECOPD inpatients. Therefore, this study investigated the association of changes in coagulation function and C-reactive protein (CRP) with PM levels in the days preceding hospitalization.

**Patients and methods:**

We reviewed the medical records of AECOPD patients admitted to Putuo Hospital, Shanghai University of Traditional Chinese Medicine, between March 2017 and September 2019. We analyzed the association of coagulation function and CRP level in AECOPD patients with PM levels in the days before hospitalization. Multivariate unconditional logistic regression analyses were used to evaluate the adjusted odds ratio (OR) and 95% confidence interval (CI) for the association of CRP data with hospitalization day. Kruskal–Wallis tests were used to evaluate mean aerodynamic diameter of ≥ 2.5 μm (PM_2.5_) exposure on the day before hospitalization; we assessed its association with changes in prothrombin time (PT) in AECOPD inpatients with different Global Initiative for Chronic Obstructive Lung Disease (GOLD) classes.

**Results:**

The peripheral blood PT of AECOPD patients with PM_2.5_ ≥ 25 mg/L on the day before hospitalization were lower than those of patients with PM_2.5_ < 25 mg/L (*t* = 2.052, *p* = 0.041). Patients with severe GOLD class exposed to greater than 25 mg/L of PM_2.5_on the day before hospitalization showed significant differences in PT (*F* = 9.683, *p* = 0.008). Peripheral blood CRP levels of AECOPD patients exposed to PM_2.5_ ≥ 25 mg/L and PM_10_ ≥ 50 mg/L on the day before hospitalization were higher than those of patients exposed to PM_2.5_ < 25 mg/L and PM_10_ < 50 mg/L (*t* = 2.008, *p* = 0.046; *t* = 2.637, *p* = 0.009). Exposure to < 25 mg/L of PM_2.5_ on the day before hospitalization was significantly associated with CRP levels (adjusted OR 1.91; 95% CI 1.101, 3.315; *p* = 0.024).

**Conclusion:**

Exposure of patients with AECOPD to high PM levels on the day before hospitalization was associated with an increased CRP level and shortened PT. Moreover, PM_2.5_ had a greater effect on CRP level and PT than mean aerodynamic diameter of ≥ 10 μm (PM_10_). AECOPD patients with severe GOLD class were more sensitive to PM_2.5_-induced shortening of PT than those with other GOLD classes.

## Introduction

Chronic obstructive pulmonary disease (COPD) is considered a heterogeneous syndrome and has been associated with abnormal inflammatory immune responses in the lungs to particulate air pollution [1]. Exposure to ambient PM increases the incidence of AECOPD events, and a higher median of fine PM_2.5_ at 48 h before the onset of symptoms has been reported [2, 3]. PM exposure leads to AECOPD mediated via oxidative stress, airway inflammation, altered airway epithelial structure, and immune dysfunction [4]. Li et al. reported an association between air pollution and COPD mortality through distributed lag non-linear models, with an increase in PM level by 10 μg/m^3^ resulting in a 1.58% increase in COPD mortality over a lag of 0–15 days [5]. It is estimated that the burden of death caused by air pollution is annually as high as 40,000 people in the UK. By 2035, health and social care expenses caused by air pollution will reach a maximum of £18.6 billion [6]. With the aging of world’s population and rapid urbanization, the burden of COPD will increase in the next years. Therefore, from the perspective of public health and policy, as well as in the context of disease and environmental pollution prevention, it is crucial to understand how the pathophysiologic mechanisms induced by ambient air pollutants might induce adverse AECOPD.

Coagulation markers are potential predictors of COPD exacerbations and increased mortality, and the coagulation cascade has bene identified as a potential therapeutic target in COPD [7]. Plasma D-dimer level and blood coagulation indicators are used to monitoring the efficacy of low-molecular-weight heparin calcium therapy to improve blood coagulation and pulmonary functions in COPD patients [8]. An association has also been observed between long-term exposure to PM and level of coagulation [9]. Liu et al. reported robust associations between the constituents of PM_2.5_ (sulfate and ammonium) and coagulation markers, with an interquartile range increase in the two parameters corresponding to increments of 23% and 20% in plasminogen activator inhibitor-1 and soluble CD40 ligand levels, respectively [10]. While previous studies have demonstrated a significant association of urban air pollution with inflammation and coagulation, there is a paucity of research on the impact of particulate matter (PM) on inflammation and coagulation in AECOPD inpatients.

Shanghai is one of the most polluted cities in China, with PM_10_ and PM_2.5_ being the main pollutants [11]. For several years, air pollution has been a severe problem in Shanghai, reaching values higher than the recommendations of the World Health Organization (WHO) guidelines [12]. For instance, from March 2017 to September 2019, the mean PM_2.5_ and PM_10_ concentrations were 35 and 58 µg/m^3^, with maximum 24-h values of 189 and 255 µg/m^3^, respectively. An epidemiological survey in Shanghai reported that particles < 0.5 μm in diameter might be responsible for the adverse effects of particulate air pollution on COPD mortality [13]. Thus, there is an urgent need to investigate and understand the relationship between exposure to air pollution and the pathogenesis of AECOPD. This cohort study investigated the association between changes in coagulation function and level of the inflammatory biomarker—CRP—with PM_2.5_ and PM_10_ exposure levels in the days before hospitalization due to AECOPD. We hypothesized that PM exposure aggravates the diseases of the respiratory system associated with inflammation by inducing vascular endothelial injury and pro-coagulation.

## Materials and methods

### Study population

We retrospectively analyzed the medical records of 446 patients with AECOPD who were admitted to the Putuo Hospital Affiliated to Shanghai University of Traditional Chinese Medicine (Shanghai, China) from March 2017 to September 2019. The inclusion criteria were as follows: classification of patients with COPD according to the Global Initiative for Chronic Obstructive Lung Disease (GOLD) guidelines and acute exacerbation, defined as suddenly worsening of clinical respiratory symptoms—especially dyspnea, cough, and purulent sputum—resulting in the need for treatment measures in addition to the existing treatment regimen [14]. The data collected included the main characteristics of patients with a complete medical history. Clinical data were collected after hospitalization. Data related to C-reactive protein (CRP) concentration, D-dimer level, thrombin time (TT), prothrombin time (PT), fibrinogen (FIB) concentration, activated partial thromboplastin time (APTT), medical and medication history, smoking and exposure to PM, body mass index (BMI), and comorbidities (coronary heart disease, hypertension, asthma, bronchiectasis, and diabetes) were collected. The exclusion criteria were as follows: patients who suffered from thromboembolic disease, taking blood-thinning drugs (e.g., warfarin or antiplatelet drugs) or steroids, or place of residence and work to be out of Putuo district (Shanghai, China) within 7 days before the onset of symptoms.

The severity level of the acute exacerbations in patients with COPD was determined according to the 2016 GOLD classification [14]. In adults, serum CRP level ≥ 10 mg/L is generally considered to be a significant marker of inflammation caused by an infectious or non-infectious cause [15]. Patients with concurrent use of statins, inhaled corticosteroids and blood-thinning drugs were defined as those whose last use of statins, inhaled corticosteroids and blood-thinning drugs was ≤ 7 days before the date of AECOPD diagnosis or those still taking these drugs on the day of AECOPD diagnosis. Passive smoking was defined as being in the same room with a smoker for at least an hour/day for 12 or more consecutive months [16].

### Air pollution and climate data

The hourly concentrations of air pollutants in Putuo district were provided by the State Environmental Protection Administration of China (Monitoring Station #1141A), and the average 24-h PM_2.5_ and PM_10_ values were calculated. According to World Health Organization recommendations, daily average PM_2.5_ exposure level > 25 μg/m^3^ and PM_10_ exposure level > 50 μg/m^3^ were defined as above average daily exposure levels [12].

### Statistical analysis

Data were statistically analyzed using IBM SPSS Statistics for Windows, version 21.0. (IBM Corp., Armonk, NY, USA). All reported *p*-values were two-sided. Pearson’s correlation analysis was used to assess the relationships between PM_2.5_/PM_10_ and the level of coagulation/inflammatory factors. Student’s *t*-tests were used to assess the differences in CRP and markers of coagulation between PM exposure levels higher and lower than the average daily exposure levels. Multivariate unconditional logistic regression analyses were used to evaluate the adjusted odds ratio (OR) and 95% confidence interval (CI) for the association of CRP level with PM exposure level before the hospitalization day. Variables were statistically significant in the multivariate logistic regression model. The adjusted GOLD classes included history of smoking, hypertension, coronary heart disease, and PM_2.5_ and PM_10_ exposure levels. The Kruskal–Wallis test or the Student’s *t*-test were used to evaluate the associations between PM_2.5_ exposure levels on the day before hospitalization and changes in PT in AECOPD patients in different demographic and clinical features, such as GOLD classes, age, gender, BMI, history of smoking, use of statins, administration of inhaled corticosteroids, and incidence of comorbidities. *p*-values < 0.05 were considered statistically significant for single comparisons.

### Ethics approval and consent to participate

The study protocol was approved by the institutional review board of Putuo Hospital, Shanghai University of Traditional Chinese Medicine (approval number: PTEC-A-2018-25-1). The study was conducted in accordance with the principles of the Declaration of Helsinki.

## Results

**Fig. 1:**
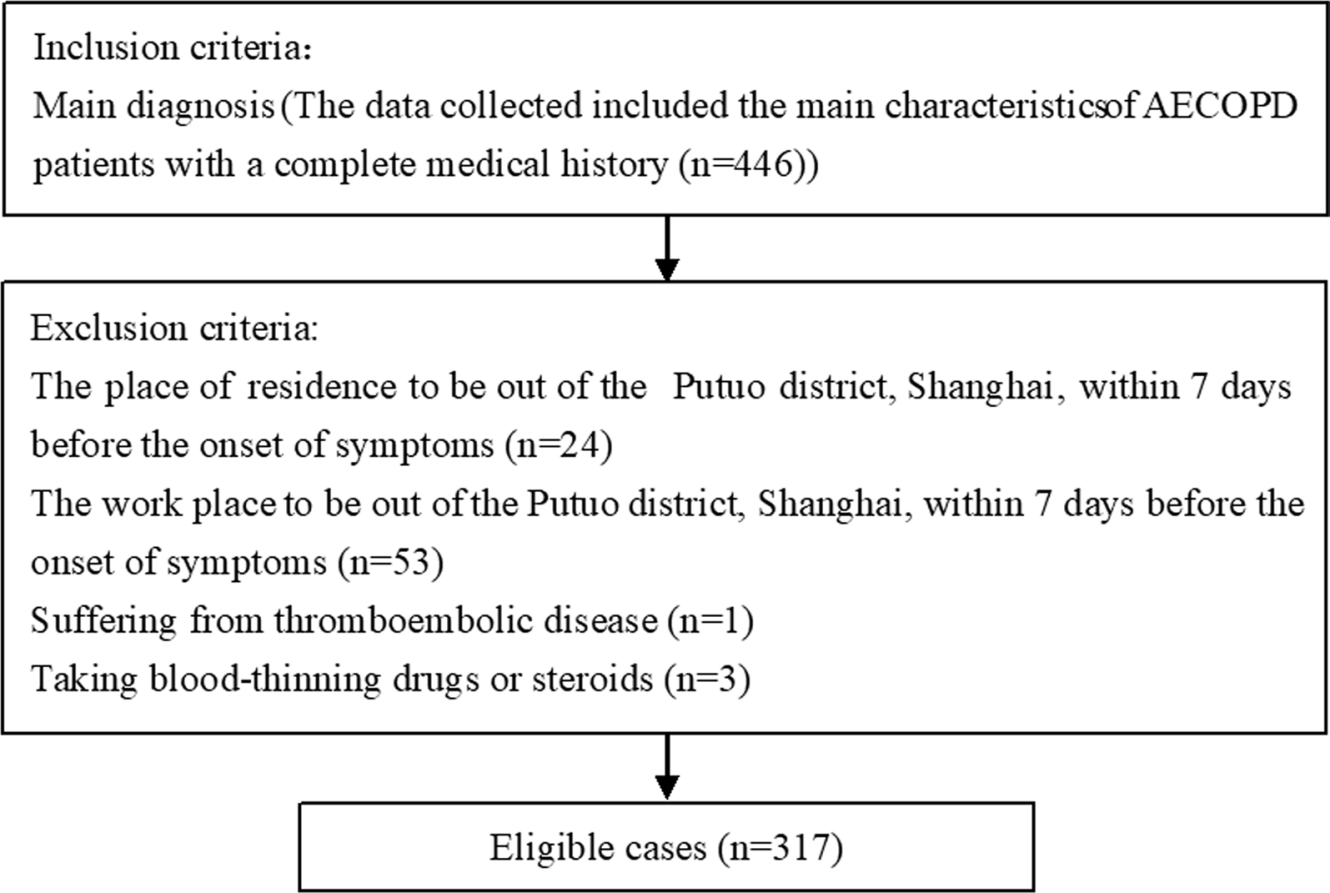
Flowchart of patients’ selection

In this study, data of 446 AECOPD patients, who were hospitalized at Putuo Hospital Affiliated to Shanghai University of Traditional Chinese Medicine between March 2017 and September 2019, were collected from medical record system and were retrospectively analyzed. Among them, 81 patients were excluded due to suffering from thromboembolic disease, taking blood-thinning drugs or steroids, or place of residence and work to be out of Putuo district within 7 days before the onset of symptoms. Finally, this study included 317 patients with AECOPD (Fig. 1). The basic demographic information of the enrolled patients (age, sex, GOLD class, inhaled corticosteroid use, smoking status, passive smoking status, statin use, inhaled corticosteroid use, and comorbidities) is shown in Table 1. The data of PM_2.5_/PM_10_ and meteorological factors in the days preceding hospitalization period are summarized in Table 2.

**Table 1:** Patients’ demographic and clinical characteristics

Variable	Description
Age years	
<65	91 (28.71%)
≥ 65	226 (71.29%)
Sex	
Female	64 (20.19%)
Male	253 (79.81%)
BMI (kg/m^2^)	
< 18.5	31 (9.78%)
≥ 18.5	286 (90.22%)
GOLD classes	
Moderate	31 (9.78%)
Severe	196 (61.83%)
Very severe	90 (28.39%)
Current smoker	
Yes	42 (13.25%)
No	275 (86.75%)
Passive smoking	
Yes	107 (33.75%)
No	210 (66.25%)
Use of statins	
Yes	58 (18.30%)
No	259 (81.7%)
Inhaled corticosteroids	
Yes	272 (85.8%)
No	45 (14.2%)
Comorbidities	
Hypertension	116 (36.59%)
Coronary heart disease	79 (24.92%)
Diabetes	46 (14.51%)
Asthma	27 (8.51%)
Bronchiectasis	7 (2.21%)

BMI, Body mass index; GOLD, Global Initiative for Chronic Obstructive Lung Disease

**Table 2:** Description of PM_2.5_/PM_10_ and meteorological factors on the days before hospitalization

Variable	Max	75th	Med	25th	Min	Mean ± SD
The day of consultation						
PM_2.5_ (μg/m^3^)	151.00	71.00	47.00	29.00	8.00	51.56 ± 27.89
PM_10_ (μg/m^3^)	144.00	76.00	56.00	38.00	5.00	58.58 ± 26.37
Temperature (°C)	34.80	27.20	17.05	8.80	1.70	18.18 ± 9.91
Relative humidity (%)	95.00	80.00	70.00	62.00	28.00	70.85 ± 12.77
The day before consultation						
PM_2.5_ (μg/m^3^)	180.00	68.00	43.00	28.00	7.00	52.32 ± 32.93
PM_10_ (μg/m^3^)	171.00	77.00	53.00	35.00	6.00	58.36 ± 29.36
Temperature (°C)	33.90	26.70	18.10	10.10	− 1.00	17.78 ± 8.87
Relative humidity (%)	98.00	81.00	73.00	64.00	28.00	71.97 ± 12.92
Two day before consultation						
PM_2.5_ (μg/m^3^)	183.00	74.00	39.00	25.00	6.00	52.05 ± 39.78
PM_10_ (μg/m^3^)	185.00	73.00	49.00	34.00	6.00	58.56 ± 35.09
Temperature (°C)	31.40	23.83	18.55	9.30	2.70	16.93 ± 8.14
Relative humidity (%)	95.00	79.00	70.00	62.00	38.00	70.80 ± 12.27

CRP, C-reactive protein; PM_2.5_, Particulate matter smaller than 2.5 µm in diameter; PM_10_, Particulate matter smaller than 10 µm in diameter

### Relationship between coagulation function in patients with AECOPD and PM level in the days before hospitalization

As shown in Table 3, the results of Pearson’s correlation analysis revealed that there were no significant relationships between PM_2.5_/PM_10_ and the levels of coagulation/inflammatory factors. When 25 mg/L was used as cutting point, no significant differences were also observed between the levels of coagulation function indicators (D-dimer level, TT, PT, FIB level, and APTT) in patients with AECOPD and the PM levels on the day and two days before hospitalization. However, the mean PT in patients with AECOPD who were subjected to PM_2.5_ level ≥ 25 mg/L on the day before hospitalization was 12.03 ± 1.89 s, which was markedly higher than that in patients subjected to PM_2.5_ level < 25 mg/L (*t* = 2.052, *p* = 0.041). The mean PT in patients with AECOPD who were subjected to PM_10_ level ≥ 50 mg/L on the day before hospitalization was 0.28 s, which was longer than that in patients exposed to PM_10_ level < 50 mg/L; however, the difference was not statistically significant (Tables 4, 5).

**Table 3:** Correlation between concentrations of PM_2.5_/PM_10_ with coagulation function and CRP

Variable	The day of hospitalization				The day before hospitalization				Two day before hospitalization			
	PM_2.5_		PM_10_		PM_2.5_		PM_10_		PM_2.5_		PM_10_	
	*R*	*p*	*r*	*p*	*r*	*p*	*r*	*p*	*r*	*p*	*r*	*p*
D dimer	− 0.027	0.627	0.004	0.943	0.016	0.775	0.043	0.453	0.010	0.855	0.033	0.557
TT	− 0.077	0.173	− 0.014	0.412	− 0.094	0.098	− 0.058	0.301	− 0.097	0.087	0.018	0.703
PT	− 0.010	0.860	− 0.026	0.639	− 0.106	0.060	− 0.096	0.092	− 0.005	0.932	− 0.082	0.150
FIB	0.010	0.858	0.003	0.985	0.054	0.341	0.023	0.832	0.045	0.424	0.032	0.572
APTT	0.047	0.404	0.066	0.251	− 0.001	0.987	− 0.047	0.409	0.014	0.804	0.005	0.953
CRP	0.050	0.405	0.078	0.184	0.068	0.248	0.083	0.162	0.011	0.855	0.047	0.428

PM_2.5_, Particulate matter smaller than 2.5 µm in diameter; PM_10_, Particulate matter smaller than 10 µm in diameter; TT, Thrombin Time; PT, Prothrombin Time; FIB, Fibrinogen; APTT, Activated Partial Thromboplastin Time; CRP, C-reactive protein

*p-v*alues are shown for the Pearson’s correlation analysis

**Table 4:** Estimated changes in coagulation function and CRP in AECOPD patients associated with PM_10_

Variable	PM_10_											
	The day of hospitalization				The day before hospitalization				Two day before hospitalization			
	< 50 mg/L	≥ 50 mg/L	*t*	*p*	< 50 mg/L	≥ 50 mg/L	*t*	*p*	< 50 mg/L	≥ 50 mg/L	*t*	*p*
	n = 158	n = 159			n = 142	n = 175			n = 133	n = 184		
D dimer	0.69 ± 0.92	0.75 ± 1.17	0.524	0.601	0.68 ± 0.92	0.75 ± 1.13	0.608	0.544	0.73 ± 1.12	0.68 ± 0.87	0.430	0.667
TT	15.99 ± 3.63	16.41 ± 1.52	1.662	0.098	16.34 ± 2.13	16.04 ± 3.45	0.949	0.343	16.38 ± 2.72	15.95 ± 2.89	1.737	0.083
PT	12.33 ± 2.35	12.31 ± 1.73	0.125	0.900	12.48 ± 1.60	12.20 ± 2.48	1.163	0.242	12.36 ± 1.85	12.30 ± 2.32	0.272	0.786
FIB	4.05 ± 2.92	3.73 ± 1.39	1.275	0.203	4.25 ± 4.01	4.53 ± 4.30	0.599	0.550	4.21 ± 3.88	4.60 ± 4.47	0.821	0.412
APTT	27.94 ± 4.58	28.81 ± 3.96	1.815	0.070	28.61 ± 4.72	28.01 ± 4.05	1.218	0.224	28.55 ± 4.12	28.09 ± 4.53	0.922	0.357
CRP	10.17 ± 12.36	10.73 ± 11.87	0.411	0.681	8.84 ± 11.54	12.41 ± 12.52	2.637	0.009	9.86 ± 12.46	11.26 ± 11.58	1.017	0.310

PM_10_, Particulate matter smaller than 10 µm in diameter; TT, Thrombin Time; PT, Prothrombin Time; FIB, Fibrinogen; APTT, Activated Partial Thromboplastin Time; CRP,C-reactive protein

*p*-values are shown for the Student’s t-tests

**Table 5:** Estimated changes in coagulation function and CRP in AECOPD patients associated with PM_2.5_

Variable	PM_2.5_											
	The day of hospitalization				The day before hospitalization				Two day before hospitalization			
	< 25 mg/L	≥ 25 mg/L	*t*	*p*	< 25 mg/L	≥ 25 mg/L	*t*	*p*	< 25 mg/L	≥ 25 mg/L	*t*	*p*
	n = 228	n = 89			n = 228	n = 89			n = 220	n = 97		
D dimer	0.75 ± 1.06	0.70 ± 1.01	0.384	0.702	0.68 ± 1.01	0.73 ± 1.03	0.690	0.399	0.69 ± 0.89	0.72 ± 1.08	0.249	0.804
TT	16.39 ± 3.47	16.13 ± 2.49	0.763	0.446	16.39 ± 2.39	16.13 ± 2.94	0.731	0.465	16.31 ± 2.24	16.16 ± 3.02	0.433	0.665
PT	12.41 ± 2.29	12.29 ± 2.07	0.450	0.653	12.61 ± 2.76	12.03 ± 1.89	2.052	0.041	12.42 ± 2.59	12.28 ± 1.90	0.550	0.583
FIB	4.33 ± 3.20	4.39 ± 4.45	0.110	0.912	4.55 ± 4.85	4.31 ± 3.83	0.466	0.642	4.29 ± 4.60	4.41 ± 3.93	0.246	0.806
APTT	27.97 ± 4.44	28.40 ± 4.35	0.770	0.442	28.13 ± 4.27	28.34 ± 4.42	0.703	0.382	28.36 ± 4.48	28.24 ± 4.32	0.229	0.819
CRP	10.52 ± 11.94	10.42 ± 12.19	0.066	0.947	8.27 ± 11.84	11.30 ± 12.13	2.008	0.046	9.33 ± 12.21	10.89 ± 12.02	1.060	0.290

PM_2.5_, Particulate matter smaller than 2.5 µm in diameter; TT, Thrombin Time; PT, Prothrombin Time; FIB, Fibrinogen; APTT, Activated Partial Thromboplastin Time; CRP,C-reactive protein

*p*-values are shown for the Student’s t-tests

### PM_2.5_ exposure level on the day before hospitalization was associated with changes in PT in patients with AECOPD with different GOLD classes

In this study present some evidence to indicate that the hypercoagulable state was affected by PM exposure in patients with AECOPD with different COPD severities (different GOLD classes). AECOPD patients with different GOLD classes exposed to PM_2.5_ levels < 25 mg/L on the day before hospitalization showed no significant difference in average PT. However, patients with AECOPD exposed to PM_2.5_ levels ≥ 25 mg/L on the day before hospitalization showed significant differences in PT according to disease severities; this is particularly true for patients with more serious AECOPD (*F* = 9.683, *p* = 0.008). There was no significant difference between PM2.5 level on the day before hospitalization and changes in PT in AECOPD patients with other different demographic and clinical features (Table 6).

**Table 6:** PM_2.5_ levels on the day before hospitalization associated with PT changes in AECOPD patients

Variable	PM_2.5_ The day before hospitalization					
	< 25 mg/L	*F/t*	*p*	≥ 25 mg/L	*F/t*	*p*
GOLD classes						
Moderate	12.57 ± 1.62	0.488	0.784	12.71 ± 1.10	9.683	0.008
Severe	12.67 ± 3.37			12.05 ± 2.10		
Very severe	12.50 ± 1.76			11.75 ± 1.47		
Age years						
< 65	12.01 ± 4.10	1.146	0.255	11.92 ± 2.03	0.627	0.531
≥ 65	12.81 ± 2.23			12.09 ± 1.83		
Sex						
Female	12.36 ± 1.00	0.406	0.686	11.77 ± 1.81	0.924	0.357
Male	12.67 ± 3.01			12.05 ± 1.88		
BMI (kg/m^2)^						
< 18.5	11.35 ± 2.43	0.946	0.347	11.62 ± 1.09	1.213	0.226
≥ 18.5	12.69 ± 2.78			12.09 ± 1.97		
Current smoker						
Yes	13.13 ± 3.70	0.450	0.654	12.01 ± 1.91	0.378	0.71
No	12.60 ± 2.72			12.14 ± 1.79		
Passive smoking						
Yes	12.46 ± 2.41	0.367	0.715	12.11 ± 1.75	0.392	0.695
No	12.69 ± 2.93			12.00 ± 2.13		
Use of statins						
Yes	13.49 ± 3.16	1.126	0.263	12.17 ± 1.71	0.441	0.659
No	12.49 ± 2.70			12.00 ± 2.49		
Inhaled corticosteroids						
Yes	12.67 ± 2.90	0.549	0.585	12.00 ± 1.76	0.570	0.569
No	12.18 ± 1.44			12.20 ± 2.51		
Comorbidities						
Hypertension						
Yes	13.11 ± 2.13	0.988	0.326	12.14 ± 1.40	0.603	0.547
No	12.26 ± 3.11			11.98 ± 2.10		
Coronary heart disease						
Yes	13.16 ± 1.82	1.108	0.271	12.15 ± 2.31	0.549	0.584
No	12.42 ± 3.01			11.99 ± 1.74		
Diabetes						
Yes	13.05 ± 1.51	0.581	0.563	12.34 ± 1.38	1.026	0.306
No	12.55 ± 2.91			11.98 ± 1.96		
Asthma						
Yes	12.84 ± 1.05	0.243	0.809	11.64 ± 1.36	0.949	0.344
No	12.59 ± 2.88			12.07 ± 1.93		

*p*-values are shown for the Kruskal–Wallis test or the Student’s t-test

PM_2.5_, Particulate matter smaller than 2.5 µm in diameter; PM_10_, Particulate matter smaller than 10 µm in diameter; BMI, Body Mass Index; GOLD, Global Initiative for Chronic Obstructive Lung Disease

### Relationship between CRP concentration in patients with AECOPD and PM exposure levels in the days before hospitalization

Similar to the changes in coagulation function indicators, patients with AECOPD showed no significant difference between peripheral blood CRP concentration and PM exposure levels on the day of and two days before hospitalization. The mean peripheral blood CRP level of patients with AECOPD exposed to PM_2.5_ levels ≥ 25 mg/L on the day before hospitalization was 11.3 mg/L—an average of 3.03 mg/L higher than that in patients exposed to PM_2.5_ levels < 25 mg/L (*t* = 2.008, *p* = 0.046). The mean peripheral blood CRP level of patients with AECOPD exposed to PM_10_ levels ≥ 50 mg/L on the day before hospitalization was 12.41 mg/L—an average of 3.75 mg/L higher than that in patients exposed to PM_10_ levels < 50 mg/L (*t* = 2.637, *p* = 0.009) (Tables 4, 5).

### Risk of increased CRP level and its association with PM exposure level on the day before hospitalization, comorbidities, BMI, smoking, and drug use

Multivariate logistic regression analysis revealed that patients with AECOPD exposed to higher PM_2.5_ levels on the day before hospitalization (PM_2.5_ ≥ 25 mg/L) had 2.19-fold higher odds of having CRP ≥ 10 mg/L when compared with those exposed to PM_2.5_ levels < 25 mg/L (95% CI 1.283–3.747) and PM_10_ < 50 mg/L (crude OR 1.86; 95% CI 1.170, 2.948). After adjusting for GOLD classes, the smoking status, hypertension, coronary heart disease, PM_2.5_, and PM_10,_ it was found that patients with AECOPD exposed to higher PM_2.5_ levels on the day before hospitalization (PM_2.5_ ≥ 25 mg/L) had 1.19-fold higher odds of having CRP > 10 mg/L when compared to those exposed to PM_2.5_ < 25 mg/L on the day before hospitalization (95% CI 1.101, 3.315), indicating a significant association of high PM_2.5_ exposure level with CRP level (*p* = 0.024). However, the adjusted OR for the risk of increased CRP and high PM_10_ exposure on the day before hospitalization was not statistically significant. GOLD class (adjusted OR 1.5; 95% CI 1.002–2.236), a current smoking status (adjusted OR 2.1; 95% CI 1.109–4.361), and hypertension (adjusted OR 1.65; 95% CI 1.019–2.683) were also significantly associated with CRP level (Table 7).

**Table 7:** PM levels on the day before hospitalization associated with CRP, incidence of comorbidities, BMI, smoking status, and administration of drugs stratified by odds ratio and 95% confidence interval

Variables	CRR		Crude		Adjusted		*p* value
	< 10 mg/L	≥ 10 mg/L	OR	95%CI	OR	95%CI	
	n = 191	n = 126					
Age years							
< 65	65 (20.50%)	36 (11.36%)	1.29	(0.791,2.103)	–	–	–
≥ 65	126 (39.75%)	90 (28.39%)					
Sex							
Female	38 (11.99%)	26 (8.20%)	1.05	(0.599,1.831)	–	–	–
Male	153 (48.26%)	100 (31.55%)					
BMI							
< 18.5 kg/m^2^	19 (5.99%)	12 (3.79%)	0.95	(0.445,2.039)	–	–	–
≥ 18.5 kg/m^2^	172 (54.26%)	114 (35.96%)					
GOLD classes							
Moderate	20 (6.31%)	11 (3.47)	0.82	(0.378,1.771)	1.5	(1.002,2.236)	0.049
Severe	126 (39.75)	70 (22.08%)	0.65	(0.406,1.023)			
Very severe	45 (14.20%)	45 (14.20%)	1.80	(1.099,2.955)			
Current smoker							
Yes	18 (5.68%)	24 (7.57)	2.26	(1.171,4.368)	2.1	(1.109,4.361)	0.024
No	173 (54.57%)	102 (32.18%)					
Passive smoking							
Yes	65 (20.50%)	42 (13.25%)	0.97	(0.602,1.560)	–	–	–
No	126 (39.75%)	84 (26.50%)					
Use of statins							
Yes	38 (11.99%)	20 (6.31%)	0.76	(0.419,1.378)	–	–	–
No	153 (48.26%)	106 (33.44%)					
Inhaled corticosteroids							
Yes	163 (51.42%)	110 (34.70%)	0.77	(0.401,1.447)	–	–	–
No	28 (8.83%)	16 (5.05%)					
Comorbidities							
Hypertension	60 (18.93%)	56 (17.67%)	1.75	(1.096,2.783)	1.65	(1.019,2.683)	0.042
Coronary heart disease	39 (12.30%)	40 (12.62%)	1.81	(1.084,3.032)	1.63	(0.952,2.774)	0.075
Diabetes	27 (8.52%)	19 (5.99%)	1.08	(0.571,2.036)	–	–	–
Asthma	12 (3.79%)	15 (4.73%)	2.02	(0.910,4.465)	–	–	–
Bronchiectasis	3 (0.95%)	4 (1.26%)	2.06	(0.452,9.340)	–	–	–
PM_2.5_							
≥ 25 ug/m^3^	126 (39.75%)	102 (32.18%)	2.19	(1.283,3.747)	1.91	(1.101,3.315)	0.021
< 25 ug/m^3^	65 (20.50%)	24 (7.57%)					
PM_10_							
≥ 50 ug/m^3^	94 (29.65%)	81 (25.56%)	1.86	(1.170,2.948)	–	–	–
< 50 ug/m^3^	97 (30.60%)	45 (14.20%)					

CRP, C-reactive protein; PM_2.5_, Particulate matter smaller than 2.5 µm in diameter; PM_10_, Particulate matter smaller than 10 µm in diameter; GOLD, Global Initiative for Chronic Obstructive Lung Disease; BMI, Body Mass Index

Variables found to be statistically significant in the multivariable unconditional logistic regression model. Adjusted for GOLD classes, current smoker, hypertension, coronary heart disease, PM_2.5_ and PM_10_

## Discussion

Air pollution is a serious worldwide problem due to its impact on the human respiratory system. Morantes et al. [3] and Wang et al. [17] have reported the association between air pollution and increased incidence of AECOPD, and the relationship between air pollution and acute respiratory inflammation has been confirmed previously [18]. Gao et al. reported that exposure to air pollutants was associated with reduced predicted forced vital capacity (FVC%pred) and increased systemic inflammation in patients with COPD, which may be attributed to changes in the proportions of CD4(+) T-cell subsets [19]. Patients with COPD are more susceptible to air pollution-associated respiratory inflammation than individuals without COPD [20]. Black carbon, a marker of exposure to traffic-based PM, has shown exposure–response relationships with biomarkers of systemic inflammation and endothelial activation in patients with COPD [21].

Increased morbidity and mortality from PM is hypothesized to be caused partly by systemic inflammation and a hypercoagulable state following pulmonary oxidative stress and inflammation [22]. However, previously reported associations for airborne particles with inflammation and hypercoagulability are highly heterogeneous. A cross-sectional study conducted by Sara et al. showed that, after adjusting for demographic, socioeconomic, behavioral, and health factors, there were limited evidences for relationships between markers of inflammation and PM_10/2.5_ or its components [23]. Another study reported that PM exposure was not accompanied with any short-term effects on PT, endogenous thrombin potential (ETP), and CRP within one week [4]. Research on the correlation among PM exposure, inflammation, and coagulation needs to eliminate a greater number of confounding factors and to consider a larger sample size, however, it is difficult to ensure that the time and concentration of exposure to PM can be unified with a larger sample size. Therefore, we are more inclined to perform research based on a small sample size and highly eliminate confounding factors. A retrospective study reported that PM_2.5_ level higher than 25 mg/L was associated with the increased purulent sputum, pleuritic chest pain, and the use of antibiotic and corticosteroid drugs [5]. We believe that PM2.5 level greater than 25 mg/L is an important cutting point for the accumulation of clinical symptoms in AECOPD patients.

Systemic biomarkers of inflammation include high blood CRP levels in cases of COPD exacerbations [24]; moreover, CRP is a predictor of COPD hospitalization and death [25]. Garshick et al. reported a positive relationship between black carbon exposure level and CRP concentration, with the largest effect size observed for black carbon levels averaged over the previous 7 days [21]. This finding reflected the hysteresis of CRP to air pollution. A meta-analysis showed that long-term exposure to particulate air pollution was more strongly associated with CRP level than short-term exposure [26]; however, CRP concentration in patients with COPD on the day of hospitalization was associated with significantly increased PM levels on the day before hospitalization, suggesting that CRP level in patients with COPD is sensitive to short-term PM_2.5_ exposure.

Even exposure to ambient PM may induce a systemic inflammatory response; however, its reported associations with CRP levels are inconsistent [26, 27]. Li et al. found no consistent relationship between PM exposure and CRP levels in adults with chronic inflammatory conditions [28]. The use of anti-inflammatory drugs may explain this reduced association. As an indicator of systemic inflammation, CRP is a marker for functional capacity and distress due to respiratory symptoms in COPD; moreover, COPD patients with very severe GOLD stage reportedly has higher CRP levels than those belonging to the other stages [29]. Statin use was associated with a reduced risk of COPD exacerbation, and statins have also been shown to independently lower CRP and reduce cardiovascular events related to hypertension [30]. In a previous study, patients with COPD included higher proportions of smokers with elevated CRP, leukocytosis, interleukin (IL)-6, and tumor necrosis factor (TNF)-α levels when compared to patients without COPD [31]. Furthermore, in this study, the mean CRP level declined with longer durations of smoking cessation. After adjusting for covariates to reduce the influence of confounding factors that induce CRP elevation, multivariate logistic regression analysis identified GOLD class, smoking status, and hypertension as the factors significantly associated with high CRP levels, consistent with the results of previous reports [32].

In this study, compared to PM_10_ exposure levels, the exposure levels of PM_2.5_ had a greater effect on peripheral blood CRP concentration. This may be due to different degrees of deposition of particulate air pollutants in the lungs, leading to different physiological and pathological changes. Large particles can enter the upper airways, while ultrafine particles can be deposited in the alveoli, translocate from sites in the lung through systemic circulation, and can easily induce systemic inflammation [33]. Inflammation and hypercoagulability are considered to be the potential mechanisms underlying PM-induced adverse cardiovascular events. As indicated by the results of this study, hypertension and coronary heart disease were identified as risk factors for increased CRP levels in patients with AECOPD, consistent with the data reported in the current literature [34].

COPD patients had a higher percentage of cardiovascular-related deaths than those without COPD [35]. With increasing air pollution, the risk of cardiovascular disease is also increasing [36]. Air pollution has been shown to elevate platelet aggregation and coagulation in healthy volunteers [27]. In addition, in patients with stable COPD, a higher D-dimer level was identified as a predictor of higher mortality, and higher levels of thrombin AT-III complex was associated with an increased risk of exacerbations in patients with COPD [37]. However, there is a lack of precise epidemiological data on the changes in coagulation function induced by air pollution in patients with AECOPD.

PT is one of the most frequently performed tests to assess global coagulation involving plasma clotting factors I, II, V, and VII [38]. Epidemiological studies have demonstrated that PM exposure shortened PT and increased the plasma level of FIB, and that these hemostatic disturbances were associated with the development of deep vein thrombosis (DVT); moreover, a higher mean PM level in the year before the examination was associated with shortened PT, increasing the risk of DVT [39]. Our study finding indicate that PM_2.5_ exposure level ≥ 25 mg/L on the day before hospitalization shortened PT in patients with AECOPD, especially in those with more severe AECOPD. This finding provides a scientific basis for the prevention of blood hypercoagulability in patients with AECOPD following short-term (i.e. on the day before hospitalization) exposure to elevated levels of air pollutants. Our findings also showed that the severe GOLD class and higher PM_2.5_ exposure levels were risk factors for increased CRP levels in patients with AECOPD. CRP may exert direct effects to aggravate endothelial dysfunction and induce augmented procoagulant responses [40]. Thus, patients with AECOPD with severe GOLD class are more sensitive to developing PM_2.5_ exposure-induced coagulation disorders. This increased sensitivity may be related to the increased CRP concentration in these patients.

PM contains volatile mixtures of numerous heavy metals, organic compounds, and carcinogens that interact with in vivo cellular mechanisms and induce vascular damage, including endothelium inflammation, development of atherosclerosis, lipid peroxidation, alterations in cytokines and acute phase protein levels (such as CRP), and procoagulant responses [41, 42]. The mechanisms by which PM accelerates disease progression in COPD involve changes in the prooxidant-antioxidant balance [43]. Air PM increases the levels of reactive oxygen species (ROS), disrupts cell homeostasis, or activates the redox-sensitive signaling pathway to activate inflammatory cells, pro-inflammatory gene expression, and cytokine production [44]. Oxidative stress markers, measured as thiobarbituric acid reactive substances, were positively correlated with the score of the COPD Modified Medical Research Council scale [29]. PM also reportedly increases ROS production, and PM exposure-induced activation of NOX-4 enzyme production in human aortic endothelial cells leads to the regulation of tissue factor mRNA, resulting in the rapid onset of thrombin generation and fibrin clot formation [45]. In another study, PM exposure induced sustained dose-dependent dysfunction of the pulmonary endothelial cell barrier via ROS-induced generation of truncated oxidized phospholipids (Tr-OxPLs) [46].

This study had several limitations. First, only few patients with mild COPD were assessed. Inpatients have a complete medical history and laboratory test results that make them highly suitable for inclusion in a retrospective analysis. However, there were fewer COPD inpatients with mild GOLD class disease or they do not meet the main criteria for the diagnosis of AECOPD. Second, although oxidative stress plays an important role in air pollution-induced airway disease exacerbation, owing to the inherent limitations of retrospective analysis, we were unable to obtain the measures of indicators of oxidative stress in hospitalized patients, which could be addressed in future studies. Third, the composition of PM remarkably varies in presence of a broad range of sources, however, national air quality monitoring stations are used to define exposure status across a large geographical area. The present study did not consider the effects of indoor air quality or domestic fuel types. Fourth, 81 (18%) patients were excluded due to incomplete data, which could make potential bias due to missing data. Our results, although preliminary, indicated that a high PM exposure level on the day before hospitalization was associated with an increased CRP level and shortened PT in patients with AECOPD. Larger prospective studies, including sequential measurements of clinical biomarkers and more granular air quality data (e.g., at residential location), are therefore required to provide a conclusive, scientific basis for the development of thromboembolic disease in patients with AECOPD with a long-term exposure to high levels of air pollutants.

## Conclusions

Collectively, our findings showed alterations in the prothrombotic state and levels of CRP in AECOPD, which were associated with short-term exposure to ambient air pollution. This study also demonstrated the greater adverse effects of PM_2.5_ exposure when compared to those of PM_10_ exposure. AECOPD patients who are subjected to high PM levels should be aware of the risk of shortened PT, especially patients in a severe GOLD class. Considering the limitations of this study, additional prospective studies should be conducted to confirm our findings.

## Data Availability

Data are available from the corresponding author upon reasonable request.

## Abbreviations

BMI: Body mass index
GOLD: Global Initiative for Chronic Obstructive Lung Disease
PM_2.5_: Particulate matter smaller than 2.5 µm in diameter
TT: Thrombin time
PT: Prothrombin time
FIB: Fibrinogen
APTT: Activated partial thromboplastin time
CRP: C-reactive protein
PM_10_: Particulate matter smaller than 10 µm in diameter
DVT: Deep vein thrombosis
ROS: Reactive oxygen species
